# Supplementary material for: Millisecond cryo-trapping by the spitrobot crystal plunger simplifies time-resolved crystallography
Source: Nat Commun. 2023 Apr 25;14:2365. doi: 10.1038/s41467-023-37834-w (PMC10130016; doi:10.1038/s41467-023-37834-w)
Supplement: Supplementary file 1 — Supplementary information [file 41467_2023_37834_MOESM1_ESM.pdf]

Supplementary Information

Millisecond cryo-trapping by the spitrobot crystal plunger simplifies time-resolved crystallography

Supplementary Notes

The *spitrobot*

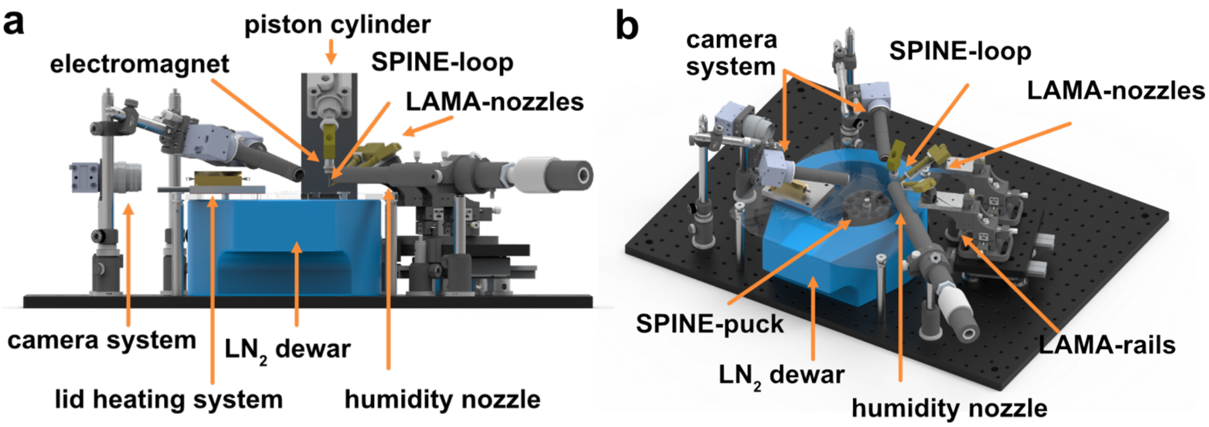

Supplementary Fig. 1: The setup of the spitrobot. *a) front view b) isometric view, the piston cylinder has been omitted for clarity. Relevant parts are labelled, please refer to the text for further details.*

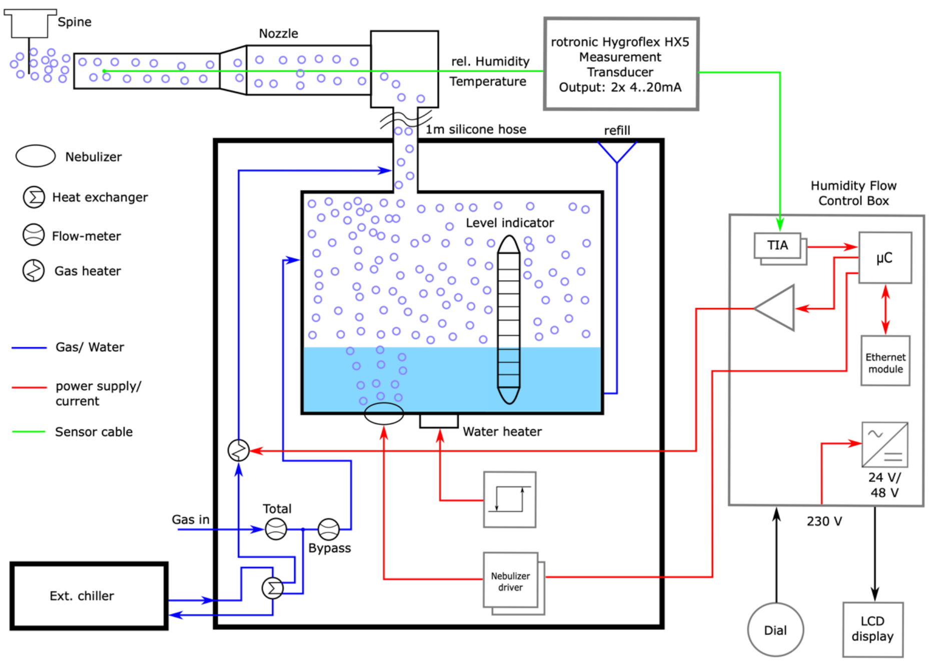

Supplementary Fig. 2: Schematic overview of the humidity flow device (HFD). *The scheme shows the interplay of the various hardware parts of the HFD.*

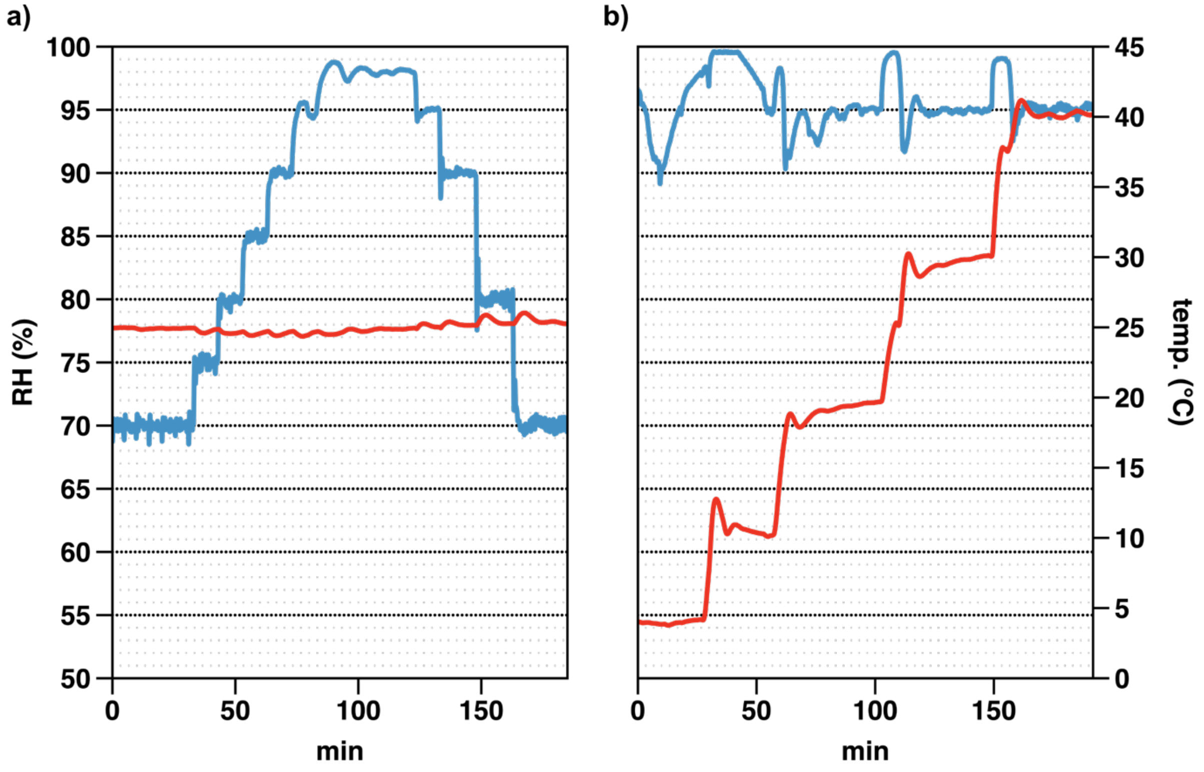

***Supplementary Fig. 3: Characterisation of the HFD**. Environmental parameters are displayed as a function of time. **a)** a step-function of the relative humidity at constant temperature (25°C). **b)** a step-function of the temperature at constant relative humidity (95%).*

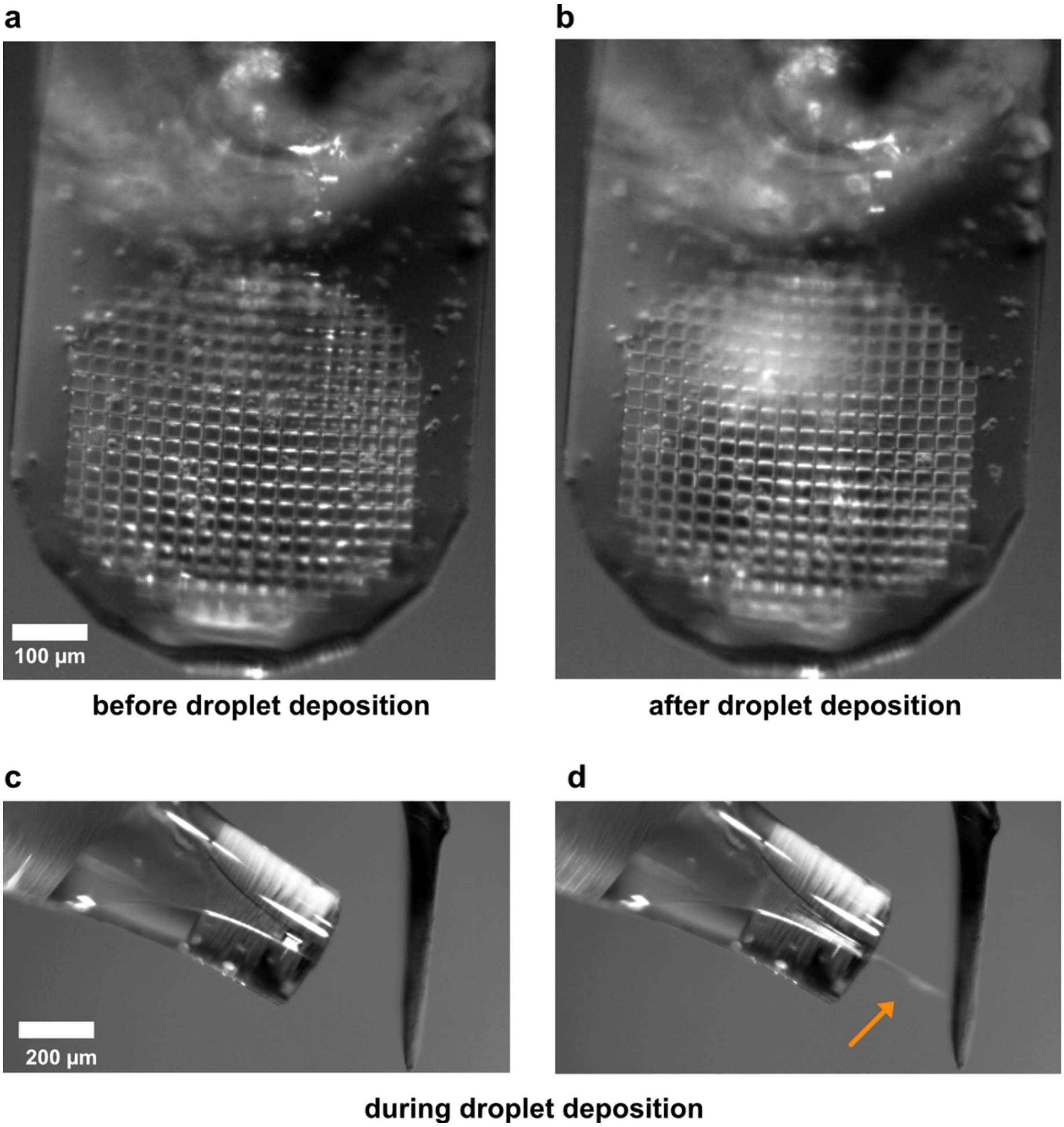

Supplementary Fig. 4: Automatic assessment of droplet deposition*. *a-b) *The figures show a comparison of a typical micro-mesh loaded with crystals a) before droplet deposition, and b) after droplet deposition. The nozzle outlet is visible in the top part of the figure, behind the semi-transparent micro-mesh material. The side view (c,d) shows the nozzle to micro-mesh distance and the droplets in flight.*

Control unit and control software

The individual steps of this protocol (reference image acquisition, droplet deposition (reaction initiation), control image acquisition, and piston motion after the pre-defined delay time) are triggered by a two-hand control device (THCD) integrated into the control unit. The two-button operation is a mandatory safety requirement to prevent inadvertent interaction with the piston during its motion, which due to its high velocity has the potential to cause serious injuries.

The delay-time settings, as well as automatic image acquisition is realized via a LabVIEW control software. Users can conveniently define file names and storage directories to obtain sample images before and after droplet deposition for quality control and referencing. In addition, users can define the delay time between droplet deposition and vitrification, which is transferred to the safety switch device. The delay time defined by the users is the total delay time for the whole period until the reaction is quenched, i.e., the time for piston motion is taken into consideration.

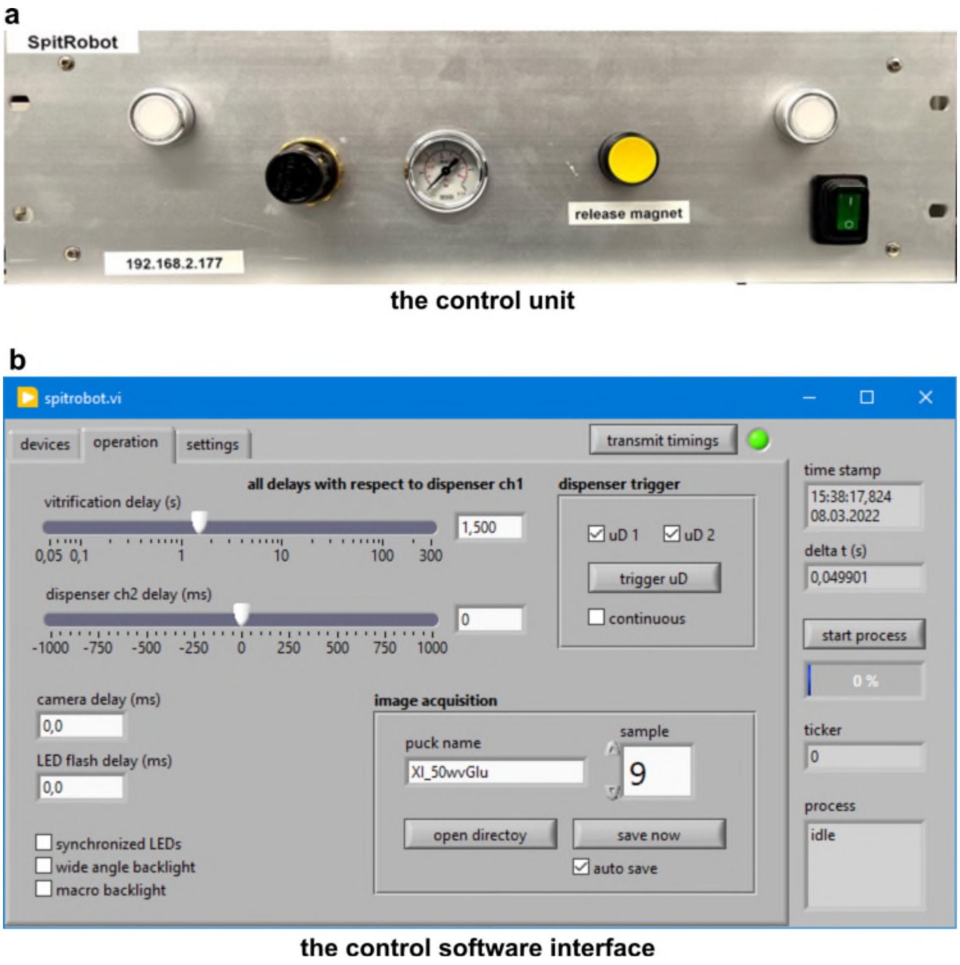

Supplementary Fig. 5: Control unit and software written in LabView. *A) The control unit is powered on via the main switch, enables to set the plunging pressure, to release the SPINE sample via an electromagnet and to start the operation cycle by simultaneously pressing the two white trigger buttons. b) via the control software all relevant parameters such as vitrification and delay times, file name and sample numbering can be set and transferred to the control unit.*

Workflow

Spitrobot preparation

The HFD should be switched on approximately 1 h before starting to load samples to allow for equilibration of temperature and humidity. Immediately prior to mounting in the spitrobot, the micromeshes are glow-discharged for 60 seconds in a glow-discharge machine, typically used for EM-grid preparation (CTA 010, Balzers Union, Switzerland). A SPINE crystal storage puck and the vitrification dewar are pre-cooled in liquid nitrogen and then transferred to the spitrobot. The LAMA nozzle is loaded with the ligand solution of interest (pre-mixed with an appropriate cryo-protectant if required). Droplet formation conditions must be established by adjusting voltage and pulse width in the Mircodrop controller (Microdrop technologies, Norderstedt, Germany). Due to the individual ligand-solution parameters, droplet distribution on a micromesh and the number of droplets must be established on a dummy sample using the camera video for visual verification. The number of droplets is set in the Microdrop controller.

Operation

Initially project directory and sample names are defined in the control software, and the desired time delay is set and transferred to the control unit. For crystal loading the LAMA nozzle is retracted to allow easier access to the sample. A micromesh sample is placed on the electromagnet to adjust camera height, position, and focus. Crystals can be loaded either by manual fishing, which works for both macroscopic crystals as well as micro-crystal slurries. Alternatively, micro-crystal slurries can be loaded via droplet deposition from a micro-pipette directly on the micromesh placed in the humidity stream. Next, an approximate nozzle position is adjusted using the manual translation stages. Using the THCD, user safety is maintained and the automated part of the spitrobot operation is started: (1) the predefined number of droplets is shot onto the micromesh, (2) after the pre-defined delay time the piston drives the sample into the liquid nitrogen where the crystal is vitrified directly in a precooled vial in the storage puck, (3) after 2 s the electromagnet releases the sample, and (4) the piston moves back into its starting position. Then the user manually rotates the storage puck into the next position to prepare the system for the next round of operation. Due this partially automated process, the complete loading of a puck with 10 samples takes only about 30 - 60 min.

Controlled crystal dehydration

To demonstrate the precise control of the humidity at the sample position and highlight the potential for controlled crystal dehydration we directly attached the humidity nozzle to the diffractometer at beamline P14 at EMBL Hamburg. We chose xylose isomerase as a model system. A macroscopic crystal of xylose isomerase was mounted in a canonical nylon loop and placed in the humidity stream at 99% relative humidity. Canonical rotation datasets were collected at consecutively lower levels of relative humidity. To mitigate potential radiation damage, the dose was kept as low as reasonably possible at an average diffraction weighted dose of 3.5 kGy per dataset[1,2]. The consecutive datasets show an overall ~20% change in unit-cell volume accompanying the reduction in relative humidity, eventually resulting in an alternate spacegroup (**Supplementary Fig. 6, Supplementary Table 1**). This also reflects in the crystal quality parameters, which surprisingly improve after the change in spacegroup, re-emphasizing the potential of controlled crystal dehydration for data-collection. The details of this observation will be discussed elsewhere. Briefly, unit-cell compactions, including xylose isomerase due to controlled crystal dehydration have been reported previously with devices operating within similar relative humidity levels (~70-99%) [3–6].

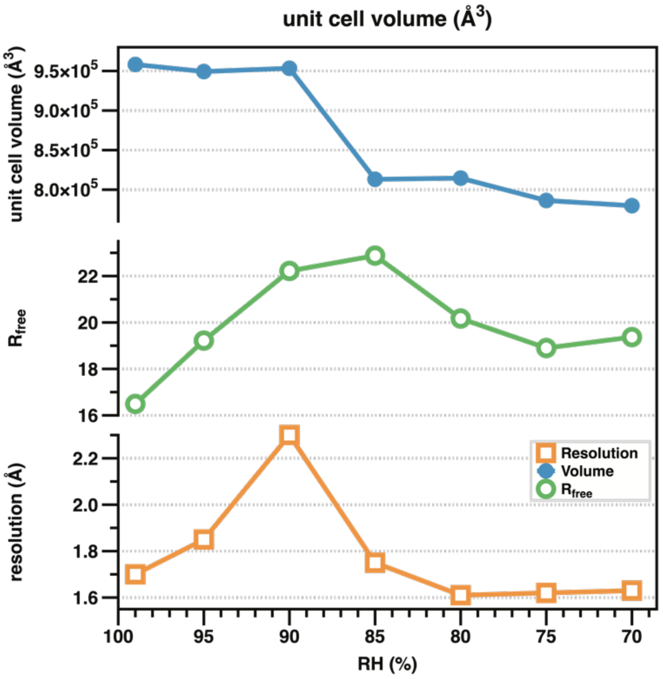

Supplementary Fig. 6: Xylose isomerase unit cell volume and selected data-quality indicators as a function of relative environmental humidity. *Resolution is depicted in orange squares, R_{free}-values are depicted in green circles, and unit cell volume is depicted in blue dots.*

Supplementary Table 1:

	XI						
PDB-ID	8AWE	8AWD	8AWB	8AWC	8AWF	8AW9	8AW8
Relative humidity	99%	95%	90%	85%	80%	75%	70%
I/σ(I)	5.2(1.6)	4.1(1.2)	6.9(2.0)	9.4(2.0)	9.9(0.7)	12.2(1.7)	11.9(1.6)
CC 1/2 (%)	99.5(71.7)	96.8(30.5)	85.9(73.7)	99.6(72.8)	99.9(31.3)	99.8(58.8)	99.9(59.3)
Completeness (%)	74.5(56.9)	99.73(99.65)	81.05(57.94)	72.5(50.6)	99.04(95.40)	67.1(25.3)	66.6(22.9)
Resolution range (Å)	46.88-1.70 (1.74 - 1.70)	49.55-1.85 (1.89 - 1.85)	69.14-2.30 (2.44 - 2.30)	68.29-1.75 (1.81 - 1.75)	43.78-1.61 (1.63 - 1.61)	52.99-1.62 (1.64 - 1.62)	52.83-1.63 (1.66 - 1.63)
R_{Free}	0.1649	0.1922	0.2222	0.2288	0.2017	0.1890	0.1937
R_{Work}	0.1358	0.1600	0.1738	0.1898	0.1787	0.1547	0.1598
Cell Dimensions a, b, c (Å); α,β,γ (°)	93.76, 99.39, 102.81 90.00, 90.00, 90.00	93.48, 9.11, 102.46 90.00, 90.00, 90.00	93.84, 99.36 , 102.26 90.00, 90.00,90.00	87.16, 94.51, 98.79 90.00, 90.00, 90.00	87.10, 98.75, 94.71 90.00, 90.00, 90.00	83.14, 96.59, 97.91 90.00, 90.00, 90.00	82.83, 96.31, 97.76 90.00, 90.00, 90.00
Space Group	I222	I222	I222	I222	P 21 21 2	P 21 21 2	P 21 21 2

Supplementary Table 2: Data collection and refinement statistics for cryo SSX crystal datasets

	XI	CTX-M-14
PDB-ID:	8AWY	8B3M
Delay time	50 ms	1000 ms
Ligand	2,3-butanediol	avibactam
**Data collection**		
Space group	I 2 2 2	P 31 2 1
Cell dimensions (a, b, c (Å))	92.7, 102.4, 99.3	41.9, 41.9, 232.9
α, β, γ (°)	90.00, 90.00, 90.00	90.00, 90.00, 120.00
Resolution (Å)	71.27 - 1.60 (1.657 - 1.60)	77.62 - 1.60 (1.66 - 1.60)
Number of images	30137	34453
I/σ(I)	9.70(5.96)	6.37 (1.83)
CC 1/2	97.92(95.19)	96.37 (73.00)
Completeness (%)	99.97 (99.98)	100 (100)
Redundancy	662.0(454.3)	532.7 (369.1)
**Refinement**		
Resolution (Å)	1.6	28.60-1.95
Total reflections	41335785	17436555
Unique reflections	62424	32733
R_{work} / R_{free}	0.1743 / 0.1940	0.2499 / 0.2968
**No. atoms**		
Protein	3111	1968
Ligand	9	17
Water	485	265
**B factors**		
Protein	13.2	24.65
Ligand	24.16	31.44
Water	27.89	30.86
**R.m.s. deviations**		
Bond lengths (Å)	0.005	0.006
Bond angles (°)	0.85	0.921
**Ramachandran statistics**		
Favored regions (%)	96.88	96.12
Allowed regions (%)	2.86	2.33
Outliers (%)	0.26	1.55

Supplementary Table 3: Data collection and refinement statistics for CTX-M-14^{E166A} single crystal datasets

	CTX-M-14		
PDB-ID:	8B2W	8B2V	8B2O
Delay time	0.5 s	1 s	5 s
**Data collection**			
Space group	P 31 2 1	P 31 2 1	P 31 2 1
Cell dimensions (a, b, c (Å))	41.81, 41.81, 232.86	41.88, 41.88,232.90	41.81, 41.81, 232.96
α, β, γ (°)	90.00, 90.00, 120.00	90.00, 90.00, 120.00	90.00, 90.00, 120.00
Resolution (Å)	77.62 – 1.89	77.64-1.73	77.65 - 1.86
R merge	0.22 (1.368)	0.24 (1.193)	0.27 (1.69)
I/σ(I)	9.5 (2.0)	9.9 (2.3)	8.8 (2.0)
CC 1/2	99.9 (71.4)	99.8 (72.4)	99.8 (71.8)
Completeness (%)	79.9 (32.2)	80.2 (36.9)	80.7 (37.2)
Redundancy	10.1 (10.0)	12.5 (10.0)	15.3 (15.4)
Number of observations	136189 (6755)	181752 (7263)	200593 (10125)
Number of unique	13533 (677)	14507 (726)	13101(657)
**Refinement**			
Resolution (Å)	36.21 - 1.78 (1.85 - 1.78)	38.82 - 1.73 (1.79 - 1.73)	38.83 - 1.89 (1.96 - 1.89)
Total reflections	27020	28951	26159
Unique reflections	13531	14503	13095
R work / R free	0.2328 / 0.2855	0.2009 / 0.2466	0.2142 / 0.2535
**No. atoms**			
Protein	1967	2004	1962
Ligand	24	24	24
Water	171	188	146
**B factors**			
Protein	21.79	22.1	27.39
Ligand	25.82	30.42	27.87
Water	25.61	30.15	34.94
**R.m.s. deviations**			
Bond lengths (Å)	0.015	0.003	0.002
Bond angles (°)	1.76	0.67	0.56
**Ramachandran statistics**			
Favored regions (%)	97.29	98.45	98.06
Allowed regions (%)	2.33	1.16	1.55
Outliers (%)	0.39	0.39	0.39

Supplementary Table 4: Data collection and refinement statistics for XI single crystal datasets

	XI			
PDB-ID:	8AWS	8AWU	8AWV	8AWX
Delay time	0.05 s	0.25 s	0.5 s	1 s
**Data collection**				
Space group	I 2 2 2	I 2 2 2	I 2 2 2	I 2 2 2
Cell dimensions (a, b, c (Å))	93.18 98.64 102.64	93.17 98.92 102.57	92.66 98.66 102.38	93.18 98.81 102.57
α, β, γ (°)	90.00, 90.00, 90.00	90.00, 90.00, 90.00	90.00, 90.00 90.00	90.00, 90.00 90.00
Resolution (Å)	68.99 - 2.26 (2.34 - 2.26)	67.82 - 1.47 (1.52 - 1.47)	51.19 - 1.58 (1.64 - 1.58)	51.29 - 1.96 (2.03 - 1.96)
R merge	0.273(2.11)	0.082(0.033)	0.144(1.433)	0.126(1.039)
I/σ(I)	8.6(1.4)	11.0(1.4)	10.6(1.6)	11.5(2.1)
CC 1/2	99.3(55.9)	99.8(59.3)	99.8(74.8)	99.8(74.8)
Completeness (%)	84.3(51.1)	93.53 (49.19)	53.34 (5.35)	85.64 (83.04)
Redundancy	12.2(11.8)	5.2(3.6)	8.67(9.91)	7.64(7.68)
Number of observations	214819	393974	297842	224568
Number of unique	17665	75423	34368	29404
**Refinement**				
Resolution (Å)	2.26	1.47	1.58	1.96
Total reflections	27111	80927	64529	40982
Unique reflections	17488	75354	34366	29381
R work / R free	0.1621/0.2233	.1514/0.1708	0.15740.1946	0.1482/0.1917
**No. atoms**				
Protein	3065	3131	3104	3173
Ligand	14	14	14	14
Water	238	522	389	376
**B factors**				
Protein	23.75	18.70	21.05	23.52
Ligand	26.29	30.63	32.72	33.68
Water	27.23	34.77	32.95	34.66
**R.m.s. deviations**				
Bond lengths (Å)	0.01	0.011	0.009	0.009
Bond angles (°)	1.12	1.23	1	1.02
**Ramachandran statistics**				
Favored regions (%)	96.61	96.88	97.14	96.61
Allowed regions (%)	3.12	2.86	2.34	3.12
Outliers (%)	0.26	0.63	0.52	0.26

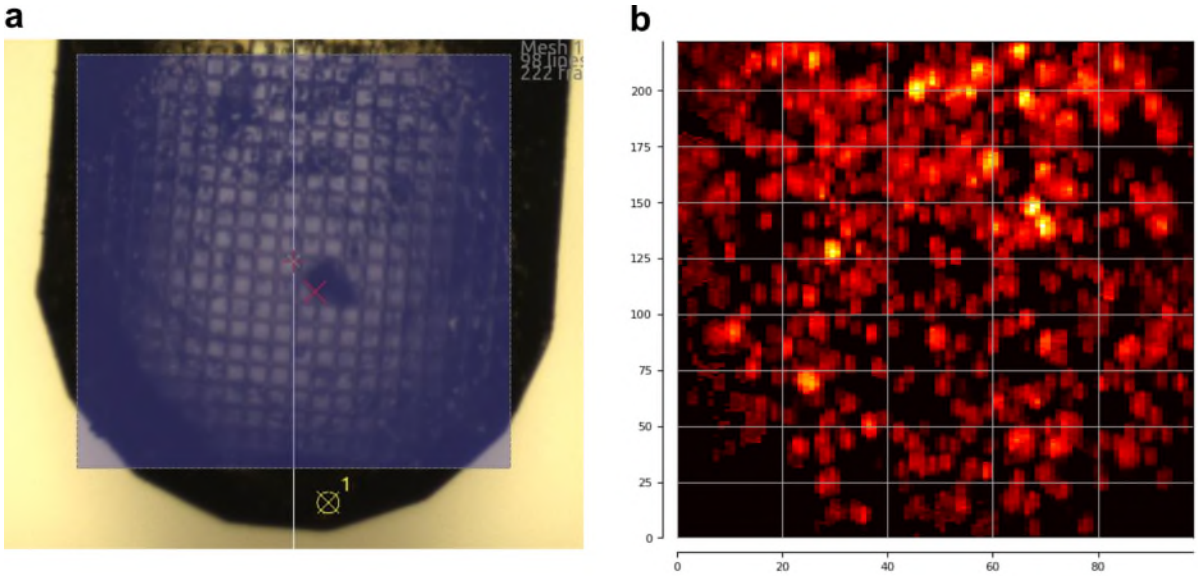

***Supplementary Fig.** 7: **Mesh heat maps**. The mesh approach can be utilized both in serial and single-crystal data collection. For cryo-SSX the heat map displays the diffraction quality of the crystals distributed on the mesh. For single-crystal data collection, the heat-maps help to identify the best diffracting crystals. a) an area on the micro-mesh is selected and raster scanned via a micro-focus X-ray beam. b) Diffraction quality results are displayed as a heat map on the same area, enabling convenient localization and selection of well-diffracting crystals. The heat map shows a typical distribution of micro-crystals on a micro-mesh.*

Cryo-trapping crystallography of tryptophane synthase reaction intermediates

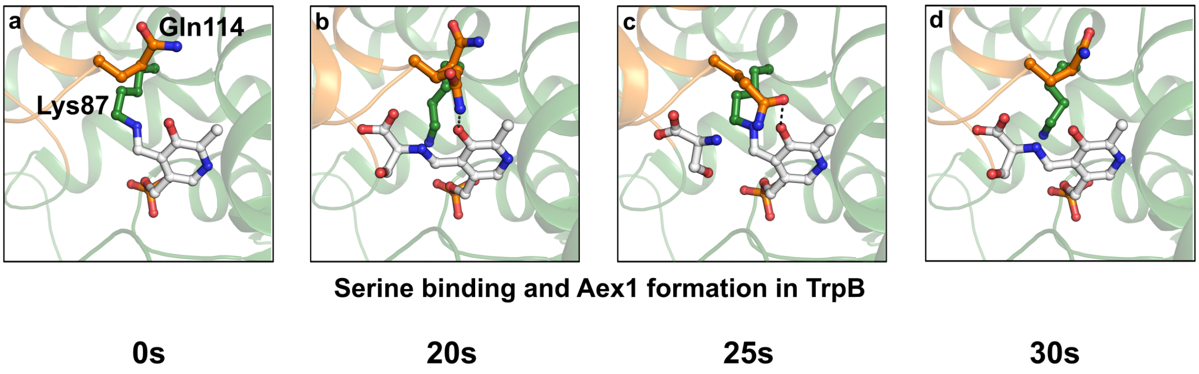

***Supplementary Fig.** 8: **TS snapshots of the residues for serine binding and Aex1 formation in TrpB, after reaction initiation.** a-d) Serine binding and Aex-Ser formation in TrpB snapshots of residues involved in an external aldimine intermediate (Aex-Ser) formation in Trp B active site at 20 s and 30 s and serine binding state at 25 s after mixing. Active site residues of TrpB and substrates are represented as ball-and-stick.*

Supplementary Table 5: **Data collection and refinement statistics for TS single crystal datasets**

	TrpSynthase			
PDB-ID:	8B03	8B05	8B06	8B08
Delay time	0 s	20 s	25 s	30 s
**Data collection**				
Space group	C 1 2 1	C 1 2 1	C 1 2 1	C 1 2 1
Cell dimensions (a, b, c (Å))	182.56, 60.44, 67.25	180.99, 59.11, 66.85	181.79, 59.05, 67.09	182.48, 60.09, 67.34
α, β, γ (°)	90.00, 94.668, 90.00	90.00, 94.598, 90.00	90.00, 94.683, 90.00	90.00, 94.746, 90.00
Resolution (Å)	2.22	2.1	2.49	2.5
R merge	0.2148 (1.41)	0.2274 (1.935)	0.2157 (1.896)	0.3221 (1.672)
I/σ(I)	8.03 (1.38)	7.62 (1.53)	7.09 (1.19)	8.21 (1.53)
CC 1/2	99.4 (73.5)	99.4 (63.9)	99.4 (55.2)	98.8 (71.6)
Completeness (%)	99.80 (99.81)	98.94 (98.91)	98.75 (98.75)	98.27 (97.62)
Redundancy	6.9 (6.9)	7.0 (7.1)	7.0 (7.0)	6.9 (7.0)
**Refinement**				
Resolution (Å)	44.15 - 2.22 (2.299 - 2.22)	43.48 - 2.1 (2.175 - 2.1)	42.47 - 2.49 (2.579 - 2.49)	44.04 - 2.5 (2.589 - 2.5)
Total reflections	249455	285705	173148	173063
Unique reflections	36333	40990	24815	25003
R work / R free	0.2111 / 0.2388	0.1904 / 0.2285	0.2323 / 0.2654	0.1966 / 0.2422
**No. atoms**				
Protein	4942	4956	4930	5001
Ligand	18	35	18	35
Water	226	239	53	137
**B factors**				
Protein	40.53	37.34	53.85	39.58
Ligand	32.85	34.73	46.86	40.97
Water	40.73	38.75	51.78	37.42
**R.m.s. deviations**				
Bond lengths (Å)	0.003	0.007	0.002	0.003
Bond angles (°)	0.62	0.89	0.49	0.62
**Ramachandran statistics**				
Favored regions (%)	97.68	97.69	97.2	95.9
Allowed regions (%)	2.16	2.31	2.65	3.64
Outliers (%)	0	0	0.16	0.46

Supplementary References:

1. Zeldin, O. B., Gerstel, M., Garman, E. F. & IUCr. RADDOSE-3D: time- and space-resolved modelling of dose in macromolecular crystallography. *J Appl Crystallogr* 46, 1225–1230 (2013).

2. Dickerson, J. L., McCubbin, P. T. N. & Garman, E. F. RADDOSE-XFEL : femtosecond time-resolved dose estimates for macromolecular X-ray free-electron laser experiments. *J Appl Crystallogr* 53, 549–560 (2020).

3. Kiefersauer, R. *et al.* A novel free-mounting system for protein crystals: Transformation and improvement of diffraction power by accurately controlled humidity changes. *J Appl Crystallogr* 33, 1223–1230 (2000).

4. Park, H. J., Tran, T., Lee, J. H., Park, H. & Disney, M. D. Controlled dehydration improves the diffraction quality of two RNA crystals. *Bmc Struct Biol* 16, 1–6 (2016).

5. Lobley, C. M. C. *et al.* A generic protocol for protein crystal dehydration using the HC1b humidity controller. *Acta Crystallogr Sect D Struct Biology* 72, 629–640 (2016).

6. Bowler, M. W. *et al.* Automation and experience of controlled crystal dehydration: Results from the European synchrotron hc1 collaboration. *Cryst Growth Des* 15, 1043–1054 (2015).
